# End-organ damage due to iron overload related to blood transfusion in an 11-year-old male

**DOI:** 10.1097/MS9.0000000000001555

**Published:** 2023-11-27

**Authors:** Ahmad Chreitah, Omar Aljanati, Fatima Bress, Bushra Jamahiri, Zeina Alkilany, Sidra Assaf

**Affiliations:** a Faculty of Human Medicine, Tishreen University; b Department of Pediatric Endocrinology Medicine, Tishreen University Hospital, Latakia, Syria

**Keywords:** autoimmune haemolytic anaemia, chelators, diabetes mellitus, iron accumulation, iron overload

## Abstract

**Introduction and importance:**

Iron overload is an abnormal accumulation of iron in parenchymal organs that leads to end-organ damage which could be either primary or secondary to repeated blood cell transfusion, its manifestations usually start in middle age and rarely in childhood.

**Case presentation:**

The authors present a rare case of an 11-year-old male with iron overload secondary to repeated packed blood transfusion for autoimmune haemolytic anaemia. He developed type 1 diabetes, pituitary atrophy, and hepatic injury. It was difficult to maintain good control of his diabetes. He had a fatal acute circulatory collapse due to multiple organ failure.

**Clinical discussion:**

Iron overload is a clinical consequence of repeated blood transfusion that could result in end-organ damage, usually occurring in adolescence and is less likely at a young age as in our case. The accumulation of iron in the tissues causes diabetes mellitus due to the destruction of β cells in the pancreas, and the increase in insulin resistance in the peripheral tissues.

**Conclusion:**

Iron overload is a serious complication of repeated blood transfusion, which could be prevented by early treatment with iron chelators at maximum tolerated doses.

## Introduction

Highlights

- Iron Overload is an abnormal accumulation of iron in parenchymal organs that lead to end-organ damage, its manifestations usually start in middle age and rarely in childhood.
- The accumulation of iron in the pancreas and other body tissues, causes diabetes mellitus due to the destruction of β cells in the pancreas, and the increase in insulin resistance in the peripheral tissues.
- Early initiation of iron chelators with the maximum tolerated doses remains the cornerstone in the management to delay the development of iron overload.

Iron overload is an abnormal accumulation of iron in parenchymal organs that leads to end-organ damage. Iron overload could be primary as hereditary hemochromatosis or secondary due to haematologic conditions with repeated packed red blood cell transfusions or ineffective erythropoiesis, liver disease, and other rare conditions such as Bantu syndrome. Clinical manifestations may include hepatic injury, pancreatic injury, heart failure, arthropathy, hypogonadism, and hyperpigmentation. The Onset of symptoms starts in middle age in males and the post-menopause period in females; it is less likely to occur in young age^1,2^. Early initiation of iron chelators might prevent life-threatening end-organ damage in the paediatric population^3^.

Autoimmune haemolytic anaemia (AIHA) is a group of disorders, characterized by the destruction of red blood cells (RBC) by autoantibodies. Based on the thermal reactivity of autoantibodies, AIHA is classified into warm AIHA, cold AIHA, and paroxysmal cold hemoglobinuria. In addition, AIHA is categorized as primary (idiopathic) or secondary based on the presence or absence of the underlying pathology^4^. AIHA in paediatrics is a relatively rare event with an estimated annual incidence of 0.8 to 1.25 cases per 100 000 children^5,6^.

Diabetes mellitus (DM) is a series of metabolic conditions associated with hyperglycaemia and caused by partial or total insulin insufficiency. There are mainly two types of diabetes; Type 1 diabetes mellitus (T1DM) is caused by autoimmune inflammatory mechanisms (positive glutamic acid decarboxylase (GAD) and undetectable levels of plasma C-peptide) leading to destruction of pancreatic beta cells that results in absolute insulin deficiency and type 2 diabetes mellitus (T2DM) is associated with b-cell dysfunction and varying degrees of insulin resistance^7^.

The accumulation of iron in the pancreas and other body tissues, such as the liver, muscles, and adipose tissue, causes diabetes mellitus due to the destruction of Beta cells in the pancreas, and the increase in insulin resistance in the peripheral tissues^8^.

We present a report of an 11-year-old male who developed an iron overload secondary to AIHA in association with Diabetes Mellitus.

This case report has been reported in line with the SCARE criteria 2020^9^.

## Case presentation

A 40-day-old baby with haemolytic anaemia, At the age of 7 months, Autoimmune haemolytic anaemia was diagnosed and treated with prednisolone, Azathioprine, and repeated packed red blood transfusion. At the age of 2 years, oral deferoxamine therapy was initiated, with elevated ferritin levels exceeding 1200 ng/ml. At the age of 11 years old, he was referred to our endocrinology unit for evaluation of poor glycemic control, and poorly controlled diabetes. He was the offspring of consanguineous parents, born at term by normal vaginal delivery. The family medical history was only positive for type 2 diabetes.

**Figure 1:**
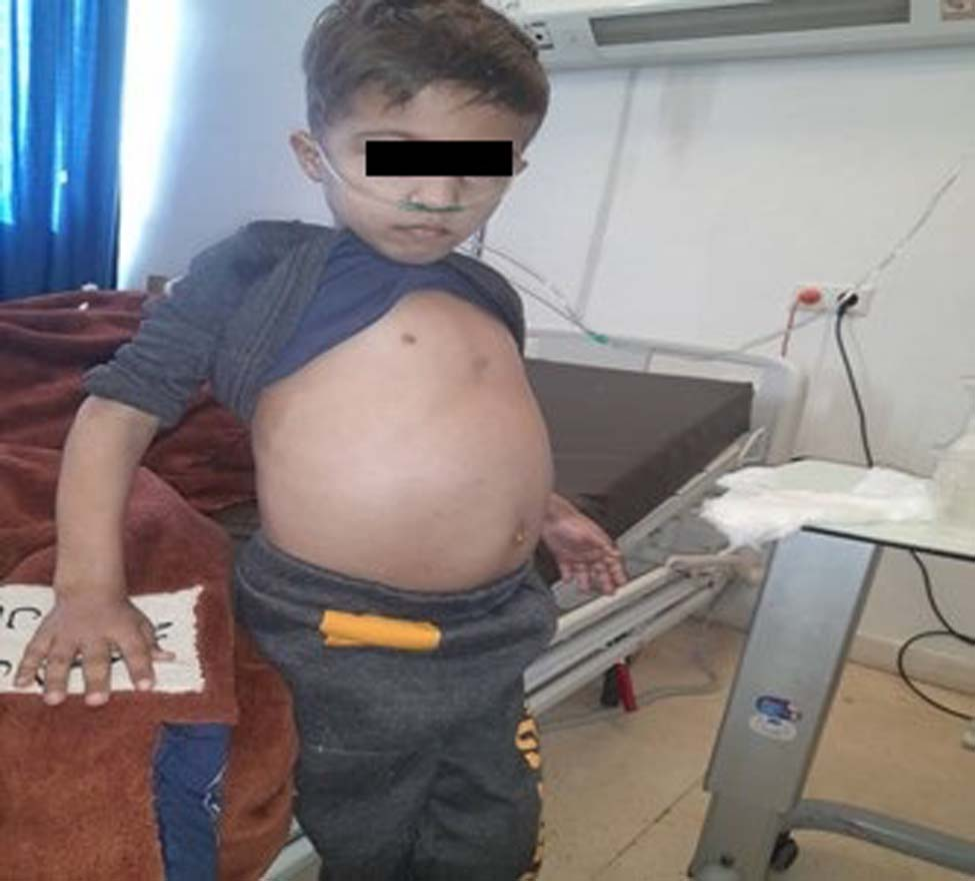
Abdominal distension and bronze skin colour.

At admission, the blood pressure was 110/70 mmHg, the heart rate of 120 bpm, and the respiratory rate of 45 bpm. On physical examination, he was conscious, weak, pale, bronze skin, with abdominal distension, and tachypnea (Fig. 1).

His weight was 21 kg (at the −3rd percentile), His height was 121 cm (below −3rd percentile), and his BMI was 14.3 kg/m2 (at the 3rd percentile) based on CDC charts. Over 11 years, he received more than 250 units of RBC transfusion.

His laboratory workup revealed an increased level of serum liver enzymes and elevated LDH with low haemoglobin value, as shown in (Table 1).

**Table 1:** The biochemical workup.

Test	Result	Normal value	Test	Result	Normal value
WBC	6.6×10^9^	4–11×10^9^ /l	MCV	82	78–100 fl
N	37%	50–70%	RDW	12	11–15%
L	57%	20–44%	PLT	380×10^9^	150–400×10^9^/l
Hb	8.8	13–18 g/dl	CRP	4	<5 mg/dl
ALT	140	10–40 U/l	Glucose	89	72–99 mg/dl
AST	69	14–20 U/l	LDH	462	110–295 U/l
TB	O,8	0.3–1.0 mg/dl	PT	12	11–13.5 s
DB	0,2	0.1–0.3 mg/dl	INR	1,3	0.8–1.1
Urea	29	15–40 mg/dl	Cr	0.7	0.5–1.0 mg/dl

ALT, alanine transaminase; AST, aspartate aminotransferase; DB, direct bilirubin; HB, haemoglobin; INR, International Normalized Ratio; L, lymphocyte; LDH, lactate dehydrogenase; MCV, mean corpuscular volume; N, neutrophil; PT, prothrombin time; TB, total bilirubin; WBC, white blood count.

The abdominal ultrasound showed splenomegaly of 13 cm and homogeneous hepatomegaly of 18 cm.

He received actrapid insulin before meals and insulin glargine at night. However, the patient had poorly controlled glucose levels despite the increased doses of Insulin to 2 units/kg per day.

A hormonal workup (Table 2) showed undetectable C-peptide levels, negative Anti-Insulin antibodies, and negative glutamic acid decarboxylase antibodies

**Table 2:** The result of hormonal findings.

Test	Result	Normal value	Test	Result	Normal value
TSH	0.62	0.5–5 miu/ul	Cholesterol	90 mg/dl	120–230 mg/dl
FT4	0.95	0.8–2 ng/dl	TG	194 mg/dl	10–140 mg/dl
ACTH	26	8–50 pg/ml	Peptide C	0 ng/ml	0.9–4 ng/ml
Cortisol	12	8–25 mcg/dl	IGF1	15 ng/dl	5–128 ng/dl
Anti-insulin Abs	2.5	Negative: up to 10 positive: more than 10	GAD Abs	2.9	Negative: up to 5 positive: more than 5

ACTH, adrenocortico trophic hormone; FT4, free thyroxine4; GAD Abs, glutamic acid decarboxylase antibodies; IGF1, insulin-like growth factor; TG, three glycerides; TSH, thyroid stimulator hormone.

Haematology and immune assessments revealed a significantly elevated ferritin level, which raise concerns about Iron Overload, as indicated in Table 3.

**Table 3:** The result of haematology and immune workup.

Test	Result	Normal value	Test	Result	Normal value
Anti-HAV	Negative	Negative	Iron	196	50–120 mcg/dl
Anti-HCV	Negative	Negative	IAT	Negative	Negative
Tuberculin ST	Negative	Negative	DAT	Negative	Negative
Procalcitonin	0.05	<0.05 μg/l	Ferritin	4877	7–140 ng/ml

Anti-HCV, anti-hepatic virus tuberculin; Anti-HAV, anti-hepatic A virus; DAT, direct anti-globulin test; IAT, indirect anti-globulin test; ST, tuberculin skin test.

**Figure 2:**
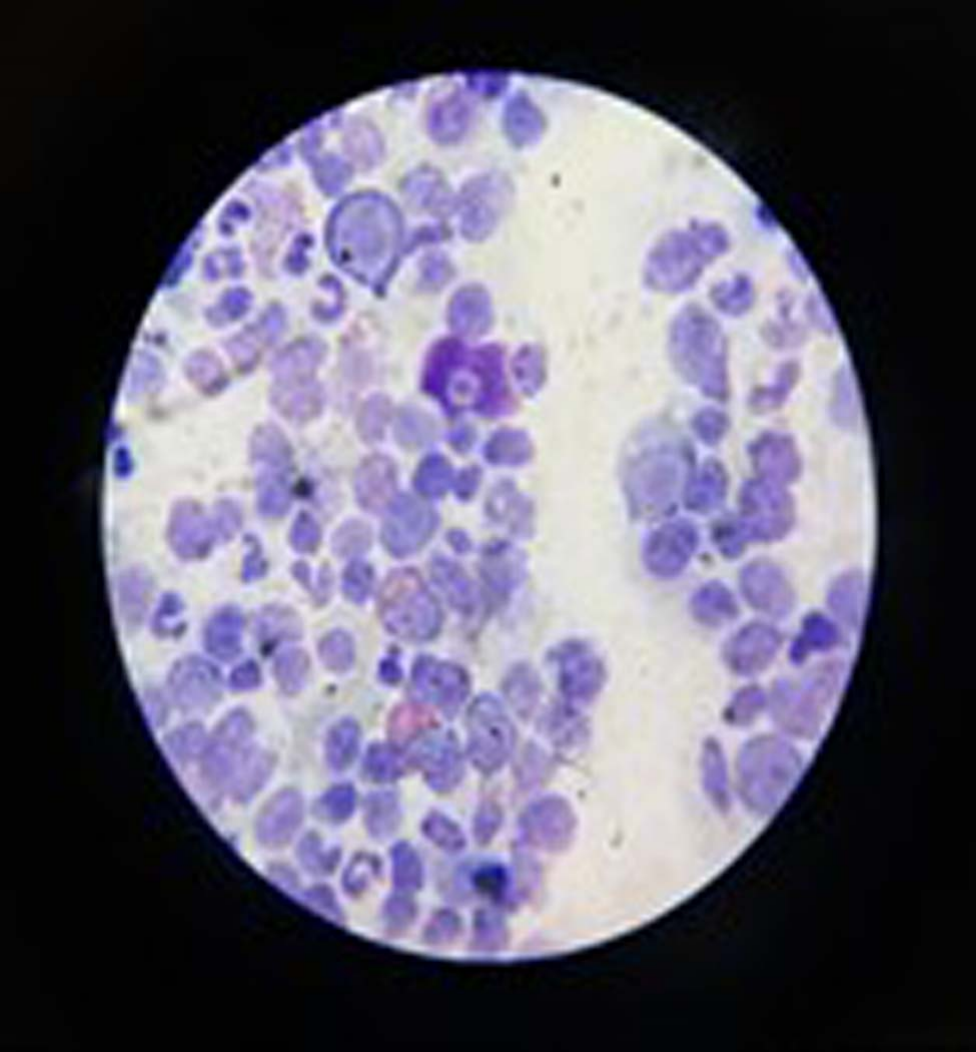
A bone marrow shows mediate cellularity, red blood cell chain hyperplasia with a relative delay of maturation, infiltration of hemosiderin granules into and out of macrophages.

A Bone marrow aspiration revealed moderate cellularity with RBC chain hyperplasia across all stages of maturation, with a relative delay in maturation observed at the acidophilic erythroblast stage. Furthermore, hemosiderin granules were noted (Fig. 2). Minimal eosinophilia was also observed.

Subcutaneous Deferoxamine was administrated at the dose of 40 mg/kg by infusion for 12 hours per day for 5 days a week with B9 (Folic acid) and B12 (cobalamin) for 5 days.

Cardiac echocardiography was normal, MRI was suggested to confirm the iron cardiac overload.

**Figure 3:**
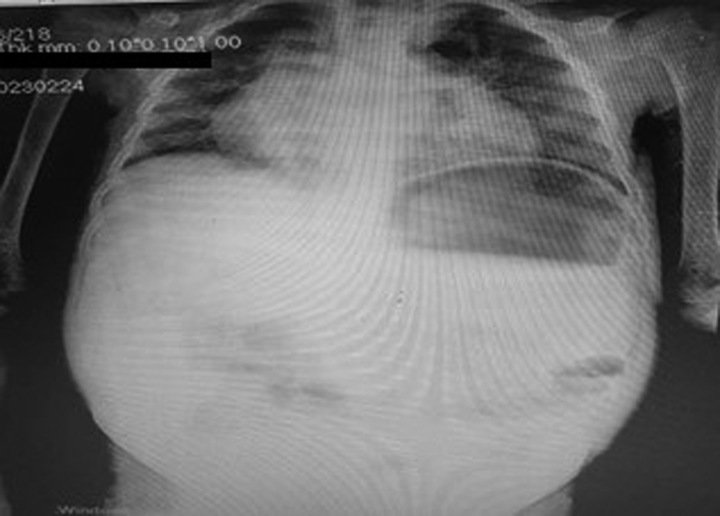
Chest X-ray showed severe bilateral infiltrates and cardiomegaly.

**Figure 4:**
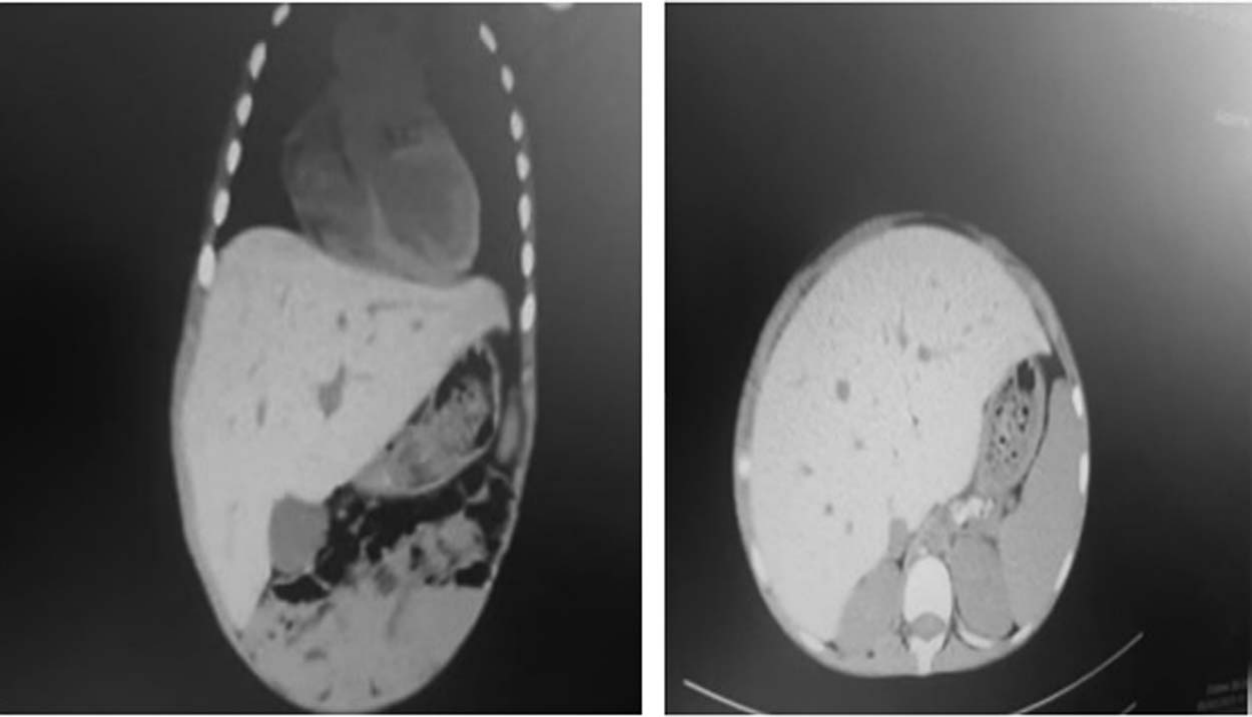
Computed tomography scan showed hepatomegaly and splenomegaly.

Due to the tachypnea and the need for oxygen; the Chest X-ray (Fig. 3) showed severe bilateral infiltration which could indicate iron accumulation, Oxygen therapy continued throughout the period of hospitalization (45 d). A full body computed tomography (CT) scan showed Alveolar densities surrounding the upper lobe of the right lung with parenchymal destruction, several dilated bronchi and bilateral mild pleural effusions, Hepatomegaly, and splenomegaly (Fig. 4).

The upper gastroscopy showed severe oedema within the gastric and duodenal mucosa, treated with intravenous omeprazole at 1 mg /kg per day for 6 weeks.

**Figure 5:**
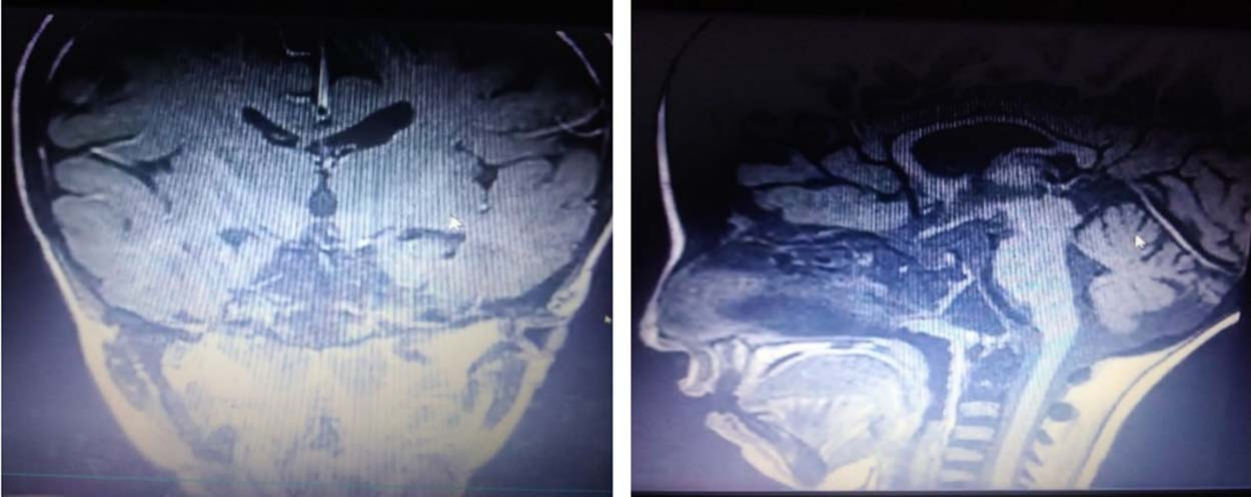
MRI Of the Pituitary gland with IV contrast showed severe atrophy of the pituitary gland.

The pituitary gland MRI with contrast showed Severe atrophy with a thickness of 1–2 mm (Fig. 5).

Ophthalmic consultation showed a mild liquefaction of the vitreous.

During his hospitalization in a paediatric surgical department for a potential splenectomy; he developed a fatal acute circulatory collapse due to multiple organ failure.

## Discussion

Autoimmune haemolytic anaemia represents a status in which the immune system attacks and destroys RBCs, resulting in intravascular or extravascular haemolysis. The management of AIHA depends on the severity of anaemia, symptoms, types of autoantibodies, and the underlying diseases which include red blood cell transfusion, pharmacotherapy (glucocorticoid, rituximab), splenectomy, IVIG, plasmapheresis, hematopoietic transplantation, and complement inhibitors. Splenectomy is an effective treatment for children with chronic or refractory primary AIHA by removing the main site of erythrocyte destruction, and also may help the patient with AIHA by removing a major site of autoantibody production. Patients undergoing splenectomy should receive appropriately timed preoperative vaccines. The patient received immunization against Streptococcus pneumoniae, Neisseria meningitides, and Hemophilus influenzae type B^10^.

Iron overload is a clinical consequence of repeated blood transfusion that could result in organ end damage^11^. In the present case, the elevated ferritin level was seen in early childhood. However, the patient had more than 250 times of red blood transfusions in his lifetime.

Iron overload is considered a chronic condition, its symptoms develop gradually over time, and the onset of its clinical manifestation occurs in the middle of age and is less likely in young age^1,2^.

Iron Overload induced by AIHA crisis in paediatrics is not well reported in the literature, Menchetti and colleagues described iron overload as a result of an AIHA crisis in a 66-year-old male patient with Evans syndrome. Ghobrial and colleagues described a refractory AIHA and iron overload related to blood transfusion in a child with a multi-visceral transplant^12,13^.

Phlebotomy is the most effective method to manage iron overload. However, since patients with inherited or acquired anaemia cannot be treated in this way, Iron Chelators remain the therapy of choice. Deferoxamine is usually administrated subcutaneously. Deferasirox is an oral iron chelator, the side effects include abdominal symptoms, skin exanthema, elevated serum creatinine, liver dysfunction, renal tubulopathy, and ulcerations^14^.

Transfusion-related iron overload in different types of congenital and acquired anaemia was successfully managed with iron chelation therapy^15,16^. However, the management does not exclude the risk of Iron overload development lately; Tariq *et al*.^17^ described a case of 52 years old American African woman with sickle cell anaemia and a history of multiple blood transfusions who developed secondary Iron Overload despite receiving phlebotomies in addition to an iron chelator.

Iron accumulates mainly in the pancreas, liver, and heart, leading to diabetes, liver cirrhosis, and heart failure. Diabetes secondary to iron overload is characterized by both insulin deficiency and insulin resistance imitating both type 1 and type 2 diabetes and is most likely caused by iron-dependent catalysis, which impairs insulin signalling in skeletal muscles and the liver and causes Beta-cell destruction due to insufficient Beta-cell antioxidant defense^18,19^. Thus, in our case, the patient developed a state of insulin resistance leading to a high dose of insulin. With negative GAD Abs and anti-Insulin Abs, which exclude autoimmune-mediated diabetes.

Iron tends to accumulate initially in Kupffer cells, leading to hepatic hemosiderin deposition. With continued transfusions, iron is eventually deposited in hepatocytes resulting in hepatocyte damage, synthetic dysfunction, fibrosis, and cirrhosis^20^.

The accumulation of iron in the pituitary gland, thyroid gland, and liver impairs the cells that play a role in the synthesis of TSH, FT4, and IGF1, with a consequent decrease in the levels of those hormones leading to Hypothyroidism and hypopituitarism, growth failure and poor sexual development^21^.

## Conclusion

The uniqueness of the case is the early development of iron overload due to repeated packed blood transfusion, which led to end-organ damage.

The possibilities of splenectomy and immune suppressors could only reduce the frequency of packed blood transfusions, but his prognostic would remain uncertain.

Early initiation of iron chelators with the maximum tolerated doses remains the cornerstone in the management to delay the development of iron overload.

Regardless of the exact underlying aetiology of his haemolytic anaemia, such a dramatic outcome should never be encountered at this young age for economic reasons. Lives of sick children all over the world should be saved according to the United Nations Convention on the Rights of children.

## Ethical approval

This case report did not require a review.

## Consent

Written informed consent was obtained from the patient for publication of this case report and accompanying images. A copy of the written consent is available for review by the Editor-in-Chief of this journal on request.

## Sources of funding

This research did not receive any specific grant from funding agencies in the public, commercial, or not-for-profit sectors.

## Author contribution

A.C.: contributed in data interpretation and as a mentor and reviewer for this case report. O.A.: contributed in data interpretation and as a mentor and reviewer for this case report. F.B.: contributed in writing the paper, data collection. B.J.: contributed in writing the paper, data collection. Z.A.: contributed in writing the paper. S.A.: contributed in writing the paper.

## Conflicts of interest disclosure

All of the authors declare that they have no competing interests.

## Research registration unique identifying number (UIN)

Not applicable.

## Guarantor

Ahmad Chreitah.

## Data availability statement

No new data were generated or analysed during this study.

## Provenance and peer review

Not commissioned, externally peer-reviewed.
